# Wound healing of experimental equine skin wounds and concurrent microbiota in wound dressings following topical propylene glycol gel treatment

**DOI:** 10.3389/fvets.2023.1294021

**Published:** 2023-12-14

**Authors:** Raphael Labens, Sharanne Raidal, Cathrine Borgen-Nielsen, Stephen Pyecroft, Sameer D. Pant, Thomas De Ridder

**Affiliations:** ^1^School of Agricultural, Environmental and Veterinary Sciences, Faculty of Science, Charles Sturt University, Wagga Wagga, NSW, Australia; ^2^QBiotics Group Ltd., Yungaburra, QLD, Australia; ^3^School of Animal and Veterinary Sciences, Faculty of Sciences, Engineering and Technology, University of Adelaide, Roseworthy, SA, Australia; ^4^Gulbali Institute, Charles Sturt University, Wagga Wagga, NSW, Australia

**Keywords:** NGS, 16S-rRNA gene sequencing, microbiome, horse, wound model, healing

## Abstract

**Introduction:**

Topical wound treatments rely on carrier formulations with little to no biological impact. The potential for a common vehicle, a propylene glycol (PG) gel, to affect wound healing measures including microbiota is not known. Microbiome characterization, based on next generation sequencing methods is typically performed on tissue or directly obtained wound fluid samples. The utility for primary wound dressings to characterize equine wound microbiota in the context of topical treatments is currently unknown. This investigation reports the topical effect of an 80% PG based gel on wound healing and microbiota in wound dressings.

**Methods:**

Experiments were performed in six mature horses utilizing a surgical, distal limb wound model, histology of sequential wound biopsies, photographic wound measurements and microbiota profiling via 16s rRNA sequencing of wound dressing samples. Experimental wounds were surveyed for 42 days and either treated (Day 7, 14, 21 and 28; at 0.03 ml/cm^2^) or unexposed to the PG gel. Wound surface area, relative and absolute microbial abundances, diversity indices and histologic parameters were analyzed in the context of the experimental group (treatment; control) using qualitative or quantitative methods depending on data characteristics.

**Results:**

Compared to controls, treatment slowed the wound healing rate (17.17 ± 4.27 vs. 18.56 ± 6.3 mm^2^/day), delayed the temporal decline of polymorphonucleated cells in wound beds and operational taxonomic units (OTU) in wound dressings and lowered alpha-diversity indices for microbiota in primary wound dressing. Relative abundances of OTUs were in line with those previously reported for equine wounds. Clinical outcomes 42 days post wounding were considered similar irrespective of PG gel exposure.

**Discussion:**

Results highlight the potential for vehicle exposure to alter relevant wound outcome measures, imposing the need for stringent experimental control measures. Primary wound dressings may represent an alternate sample source for characterization of the wound microbiome alleviating the need for additional interventions. Further studies are warranted to contrast the microbiome in wound dressings against that present on wound surfaces to conclude on the validity of this approach.

## 1 Introduction

Over the past decade and facilitated by advances in DNA detection and identification, the importance of microbiota in health and disease has increasingly been recognized (1). This progress has also highlighted the role of bacterial communities in chronic wound healing of people (2–6). Consequently, the potential for topical interventions or products to modulate microbiota and improve outcomes has been explored (7–11) but determinants of microbial communities remain poorly understood (6).

In the context of veterinary care of companion animals, further knowledge is required as comparatively few studies have evaluated the role of wound microbiota or topical interventions using culture independent methods (12–15). The equine distal limb wound model, known for its translational relevance in part due to the potential of delayed healing, facilitates research outcomes of broad application (16).

Propylene glycol (PG) an excipient of ubiquitous use in modern day life, also plays a significant role in the composition of cosmetic and pharmaceutical preparations. Being highly water soluble, its dermal absorption is limited. However, when the stratum corneum is removed as in wounds or burns, systemic absorption is facilitated leading in extreme cases to significant lactate conversion and metabolic acidosis (17, 18). Generally recognized as safe by the Food and Drug Administration it is included on the list of inactive ingredients of approved drug products (19). While detrimental effects have been observed in human keratinocyte and fibroblast cell cultures at PG concentrations below those of many product formulations (20), related negative wound healing outcomes are generally not observed. In line with the expected safety profile of PG, a 25% PG based hydrogel has not altered the rate of wound healing in a previous report using a similar equine model (21). However, the effect of any such preparation on microbiota in the local wound environment is currently unknown and may be of clinical utility. Consequently, it was hypothesized that an 80% PG based gel applied to the surface of experimental equine wounds would also not alter the healing rate but would exert a measurable effect on microbial communities. A secondary aim of this investigation was to document microbiota recovery from exudate soaked primary wound dressings and to compare results with prior studies in horses (12). As the detection of microbial communities depend on the wound sampling method (22), analyses of primary wound dressings may afford a practical and less invasive alternative.

## 2 Materials and methods

### 2.1 Animals

Four Standardbred and two Thoroughbred horses (three mares, three geldings) were recruited from the institutional teaching and research herd based on their demeanor and ease of handling. Horses’ age (mean ± standard deviation, SD) was 8.25 ± 2.96 years and body weight 512.3 ± 67.5 kg. Power analysis, based on one-way ANOVA and single post-hoc group comparisons, determined that this number of horses would allow discrimination of wound size differences ≥25%, assuming a standard deviation of 20% (*p* < 0.05; > 0.80 power).

Veterinary assessment determined that horses were free of relevant clinical abnormalities and had not received any medication within 2 weeks of the trial commencing. Horses were housed in stables (4 × 4 meters) for the duration of the study and fed a maintenance ration (hay and grain) with free access to water. Twice daily, stables were cleaned, physical examinations performed and bandages checked for their integrity. Approval for the conduct of this study was granted by the institutional Animal Ethics Committee (A18081).

### 2.2 Wound models

Following a standard intravenous anesthetic protocol for premedication (acepomazine, 0.04 mg/kg; xylazine, 1.1 mg/kg), induction (diazepam, 0.1 mg/kg; ketamine; 1.1 mg/kg) and maintenance (“triple drip”; 1,000 mL guaifenesin 5% + 500 mg xylazine +1,000 mg ketamine; maximum rate of 1 mL/kg/h) horses were placed in dorsal recumbency and the dorsal aspect of the metacarpi and metatarsi prepared for aseptic surgery (removal of hair; 5-min scrub with 2% chlorhexidine gluconate soap; isopropyl alcohol rinse). Using a template, three circular full thickness skin wounds of 3 cm diameter were sharply created and evenly spaced over the dorsum of each metacarpus/tarsus. Legs were then bandaged as outlined below and horses recovered from general anesthesia.

### 2.3 Wound management and treatments

Following creation and on each consecutive follow-up, wounds were covered with a 5×5cm hydrocellular wound dressing (Allevyn®, Smith+Nephew Inc., North Ryde, Australia), cotton wool, brown gauze bandage and an elastic adhesive bandage extending from the pastern to the level of the carpus/tarsus. The edges of the wound dressings were superglued to the surrounding intact skin to optimize stable positioning throughout a bandage’s lifespan. Whenever necessary bandages were repaired while preserving inner layers and completely changed on Day 2, 7, 14, 20, 21 and 28 after which legs remained uncovered until the end of the study on Day 42.

In each horse, limbs were randomly assigned to one of four treatment groups (negative and positive control group, i.e., no treatment or treatment with a PG (propane-1,2-diol) gel formulation; non-disclosed wound therapeutic N^o^1; nondisclosed wound therapeutic N^o^2). Here, only negative and positive control data are being reported. The PG based carrier gel, composed of 80% PG, hydroxypropyl methylcellulose and a buffering agent was applied to wounds on Day 7, 14, 21, 28 and 35 using a gloved finger at a dose rate of 0.03 mL/cm^2^ of wound area. This area was determined on the basis of the following measurements and formula: $\left(\frac{a}{2}x\frac{b}{2}\right)\times \pi$ (a = proximodistal; b = lateromedial wound dimension). If present, exuberant granulation tissue was trimmed level with the surrounding epithelial wound margin on Day 20. A volumetric estimate for excised tissue was obtained by gently compacting material inside a disposable syringe casing.

### 2.4 Outcome variables

#### 2.4.1 Wound healing

Wounds were photographed using a laser assisted wound measurement device (SilhouetteStar camera, model 2000.01, Aranz Medical, Christchurch, New Zealand) and the two-dimensional wound surface area (mm^2^) was determined by tracing wound margins in the supporting software package (Silhouette Connect). Correct camera operation included standardized calibration procedures and use of external reference points for scaling purposes. At each time point (Day 0, 2, 7, 14, 21, 28, 35 and 42) wounds were imaged three times to produce an average measure of the two-dimensional wound area. The rate of healing (mm^2^/day) was derived by subtracting wound size on Day 42 from that of Day 0 and dividing it by 42.

#### 2.4.2 Microbiotia of wound dressings

Evaluation of wound microbiome was performed on samples of exudate soaked wound dressings. For this a 4 mm leather punch was used to remove two identical disks of dressing material from the most representative area at the time of bandage change on Day 7 (pre-treatment), 14, 20 and 28. For each collection process a decontaminated punch and work surface was used. For decontamination, utensils underwent a detergent wash and ethanol rinse. The duration of sample handling (from dressing removal to placement of duplicate samples into an ethanol-dry ice slurry) was recorded to ensure samples were handled in a consistent manner. Samples were stored at-80oC for later microbiome analysis using standard DNA extraction (DNeasy PowerSoil Pro Kit, Qiagen, Melbourne Australia) and microbial profiling (Australian Genome Research Facility, Melbourne, Victoria, Australia). From each dressing only one randomly selected sample underwent processing. Microbial diversity profiling was based on partial sequencing of bacterial 16 s rRNA gene amplicons (V1 forward primer (27F): AGAGTTTGATCMTGGCTCAG; V3 reverse primer (519R): GWATTACCGCGGCKGCTG, read length 300 bp). The bioinformatics analysis involved demultiplexing, quality control, OTU clustering, and taxonomic classification. Amplicons from the primary PCR were indexed by secondary PCR and resultant amplicons were quantified by fluorometry (minimum requirement 0.2 ng/ul) and normalized. The equimolar pool was measured by qPCR and sequenced (Illumina MiSeq; San Diego, CA, USA). Paired-end reads were assembled by aligning forward and reverse reads using PEAR (version 0.9.5) (23). Primers were identified and trimmed using Seqtk (version 1.0^1^) and trimmed sequences were processed using Quantitative Insights into Microbial Ecology (QIIME 1.8.4) (24), USEARCH (version 8.0.1623) (25, 26) and UPARSE (26) software. Sequences were clustered using “rdp gold” database (27) as the reference and each read was mapped back to an operational taxonomic unit (OTU) with a minimum identity of 97% using Qiime taxonomy and Greengenes database (version 13_8, Aug 2013) (28). Relative abundances were calculated from the number of reads for each OTU relative to the total number of reads per sample. “Number of observed species,” “Chao1” richness estimator and “Phylogenetic diversity (PD) whole tree” were used for characterization of alpha-diversity. Beta diversity and the similarity in community membership and structure was visualized by principal coordinate analysis of weighted unifrac distances of microbial communities in Emperor (29) and coordinate values exported for statistical analysis.

#### 2.4.3 Wound gel characterization

Three random wound gel samples from the same batch as experimental formulations underwent microbial culture and next generation sequencing to assess the potential for iatrogenic wound contamination. Standard methods for aerobic and anaerobic bacterial culture were used and agar plates were read by sight by an experienced microbiologist. Recovered bacterial colonies were identified by colony morphology, Gram stain (Australian Biostains) morphology, catalase (3% hydrogen peroxide) and oxidase (Oxidase Strips MB0266A) testing. RNA extraction and next generation sequencing was performed as outlined in 2.4.1.2.

#### 2.4.4 Histology

A central and peripheral 6 mm punch biopsy was obtained from distal limb wounds on Day 7, 14, 20 and 28 representing separate areas of granulation tissue and epithelial wound margin. On Day 42 only central tissue samples representative of the epithelialized wound bed were collected. Sampling was facilitated by intravenous sedation (detomidine and butorphanol, 0.01 mg/kg) and locoregional anesthesia (subcutaneous 1–2 mL of 2% lidocaine hydrochloride). Samples were fixed in 10% buffered formalin for 24 h prior to embedding in paraffin for standard processing, staining (hematoxylin/eosin, gram twort, masson trichrome and picro-sirius red stains) and histologic evaluation by a veterinary pathologist blinded to experimental treatment groups (SP). Presence of polymorphnucleated cells (PMNCs) was subjectively graded as scant (up to approx 10% of observed cells), mild (up to approximately 30% of observed cells), moderate (up to approx. 60% of observed cells) or marked (any greater proportion). The proportion of immature vs. mature vascular and collagen components was estimated based on review of relevant stains and the entire area of interest. Given the topical nature of wound treatments quantitative histopathological data was collected for superficial tissue layers only. These areas were defined as extending from the clearly identifiable epidermis to one third the depth of the overall specimen thickness.

### 2.5 Statistical analyses

Wound size data from Day 0, 2, 7, 14, 21, 28, 35 and 42 was normalized using natural log transformation. In mixed model analysis of variance (ANOVA) the effect of “Time” (Day 0–42; repeated variable), “Treatment” (Nil; Gel) and “Horse” (H1-6) was assessed and this analysis repeated with “Horse” declared a random effect.

The co-dependence of wound size on the number of total OTU sequence reads was first assessed by Pearson correlation and then in separate analysis of covariance (ANCOVA) models with “Horse,” “Time” and “Treatment” as covariable to evaluate model fit. Given the availability of concurrent data, time points were limited to Day 7, 14, 20/21 and 28.

The number of total OTU reads was further correlated with “Time” (Pearson) and also evaluated using ANCOVA models with inclusion of another covariable (“Horse”; “Treatment”). Alpha-diversity data from all time points were pooled and the effect of “Horse” and “Treatment” assessed using Kruskal-Wallis and Dunn’s *post hoc* tests. Effect of “Time” was determined pooling across “Treatment” and “Horse” using Friedman and Conover’s *post hoc* tests.

Linear discriminate analysis of the three main weighted unifrac principal coordinate values (measure of beta diversity) was performed using the 4 nearest neighbors as a classification criterion. The probability to exceed the Mahalanobis distance for squared distance to “Time,” “Horse” and “Treatment” was evaluated.

Where appropriate differences of least squares means were calculated and adjusted for multiple comparisons according to Tukey–Kramer. Bonferroni adjustments were performed to Conover’s and Dunn’s *post hoc* tests. Test assumptions were assessed using Shapiro–Wilk on initial, transformed and residual data. Residual plots were inspected to verify distributions and to evaluate for heteroscedasticity.

For each treatment group histopathology data was pooled across animals and the treatment effect investigated using Mann Whitney U tests. The effect of time (i.e., biopsy sample number) was evaluated in Friedman tests and Conover *post hoc* analyses with Bonferroni adjustments. Tests were performed using SAS® on Demand for Academics (Cary, NC) or JASP (University of Amsterdam) with significance set at *p* ≤ 0.05.

## 3 Results

Data from proximal limb wounds were excluded as repeated bandage failure exposed treatment areas confounding observations. Middle wounds gave rise to observations on wound healing and microbiota. Distal wounds generated histologic data. Estimates for resected hyper granulation tissue on Day 20 and including all wounds were: H1 1.5 mL; H2 3.1 mL; H3 0.9 mL; H4 22ml; H5 and H6 1.7 mL.

### 3.1 Wound healing

**Figure 1:**
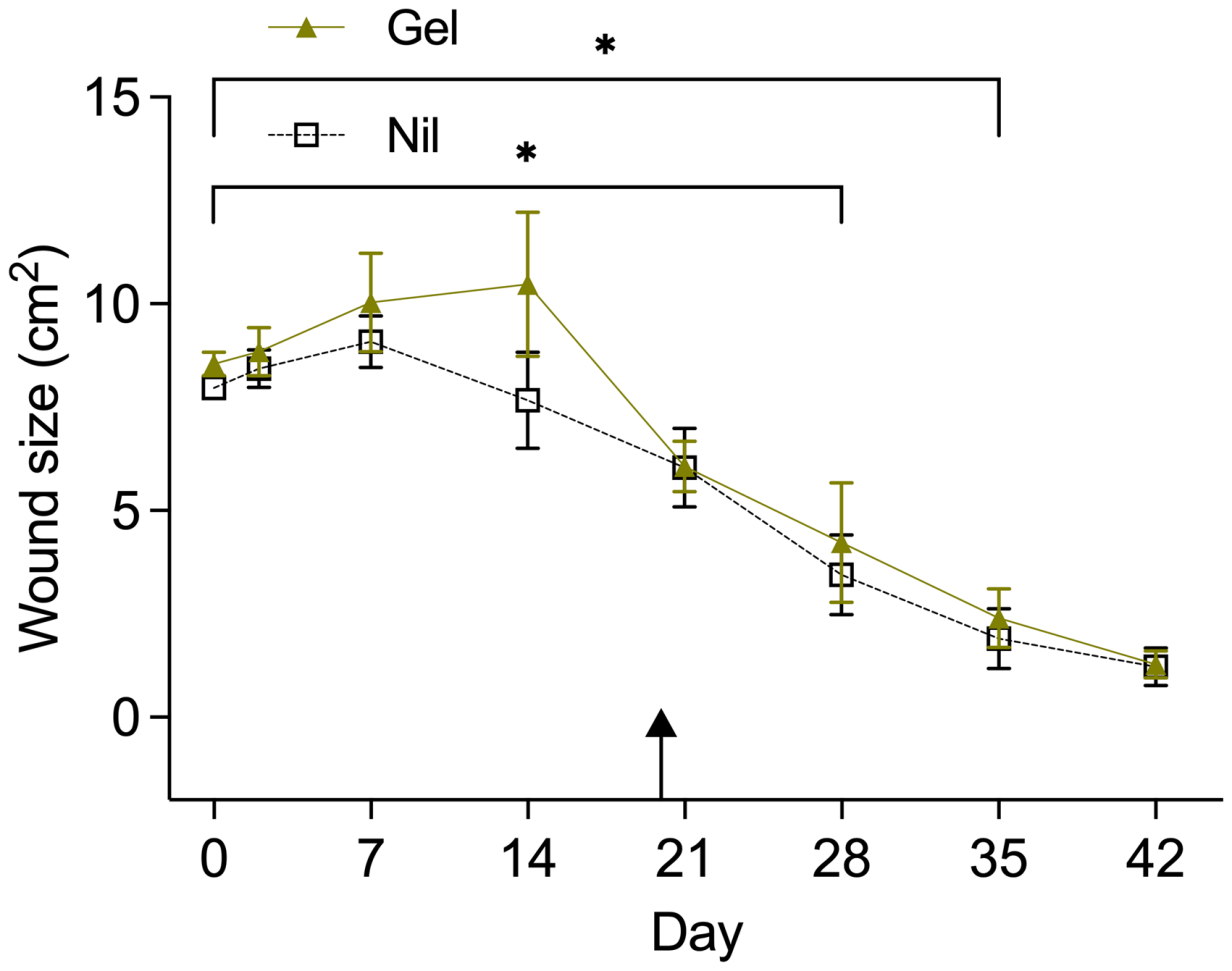
Wound area (cm^2^) from Day 0 to Day 42 by treatment (Tx). The asterix indicates a significantly smaller wound size in comparisons with Day 0 when wounds were treated (Gel) or untreated (Nil). Untreated wounds reached a smaller wound size faster (Day 28) compared to treated wounds (Day 35). Results are shown as Mean ± SEM. The arrow indicates when hypergranulation tissue was resected.

**Figure 2:**
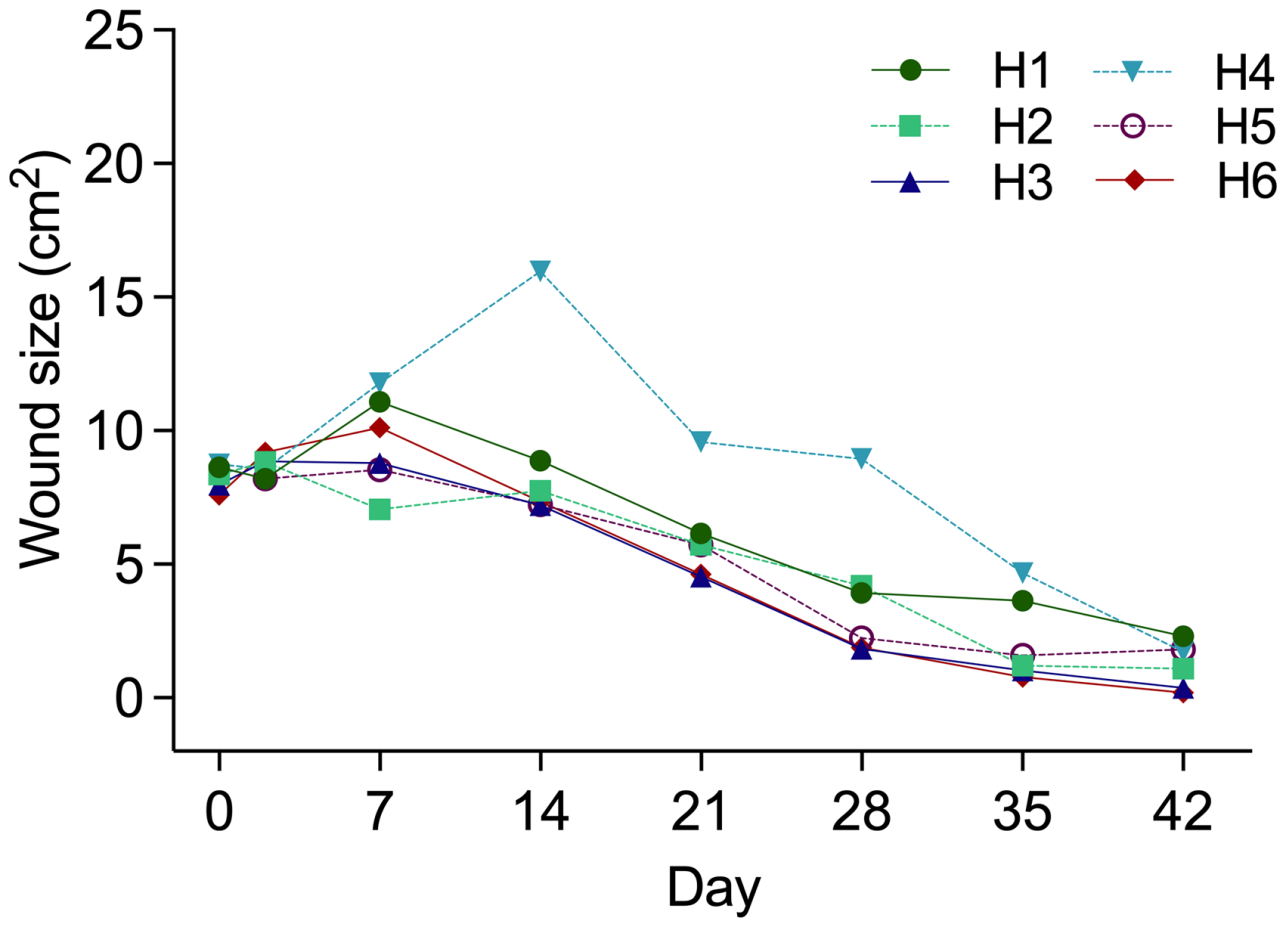
Wound area (cm^2^) from Day 0 to Day 42 by “Horse” (Number). Horse 4 illustrated distinctly larger wounds from Day 14–28. Wound debridement of excess granulation tissue occurred on Day 20. Results are shown as Mean ± SEM.

Temporal changes in wound size (cm^2^) by “Treatment” and “Horse” are visualized in Figures 1, 2 respectively. “Horse” (*p* < 0.0001), “Time” (*p* < 0.0001) and “Treatment” (*p* = 0.0126) exerted a significant effect; “Horse” was subsequently declared a random variable with no effect on the influence of “Time” and “Treatment.” Gel treated wounds were predicted to be 1.32 ± 1.1 mm^2^ larger than untreated wounds (back-transformed LSMEANS ± SEM). Compared to Day 0 gel treated wounds were significantly smaller by Day 35 (*p* = 0.0004), while untreated wounds reached significance levels sooner (Day 28; *p* = 0.0328). Average ± SD healing rates (mm^2^/day) for treated and untreated wounds were 18.56 ± 6.3 and 17.17 ± 4.27, respectively.

Wound size was positively correlated with total number of OTU reads in bandages (*R*^2^ = 0.304; *p* = 0.0356) and this association was better explained by “Horse” (*p* = 0.002; *R*^2^ = 0.459) than “Time” (*p* = 0.009; *R*^2^ = 0.345). “Treatment” exerted no explanatory function (*p* = 0.11).

### 3.2 Microbiota of wound dressings

#### 3.2.1 Absolute and relative abundances

##### 3.2.1.1 Absolute abundances

In total, 538 different OTUs were identified. The total number of OTU reads (2,080,482 in 48 samples) was negatively correlated with time (*R*^2^ = -0.544; *p* < 0.0001). This codependence was better explained by “Treatment” (*R*^2^ = 0.421; *p* = <0.0001) than “Horse” (*R*^2^ = 0.432; *p* = 0.019, ANCOVA). Each day OTU reads declined by 984 for wound dressings from gel treated wounds and by 1,603 for wound dressings from untreated wounds.

##### 3.2.1.2 Relative abundances

**Figure 3:**
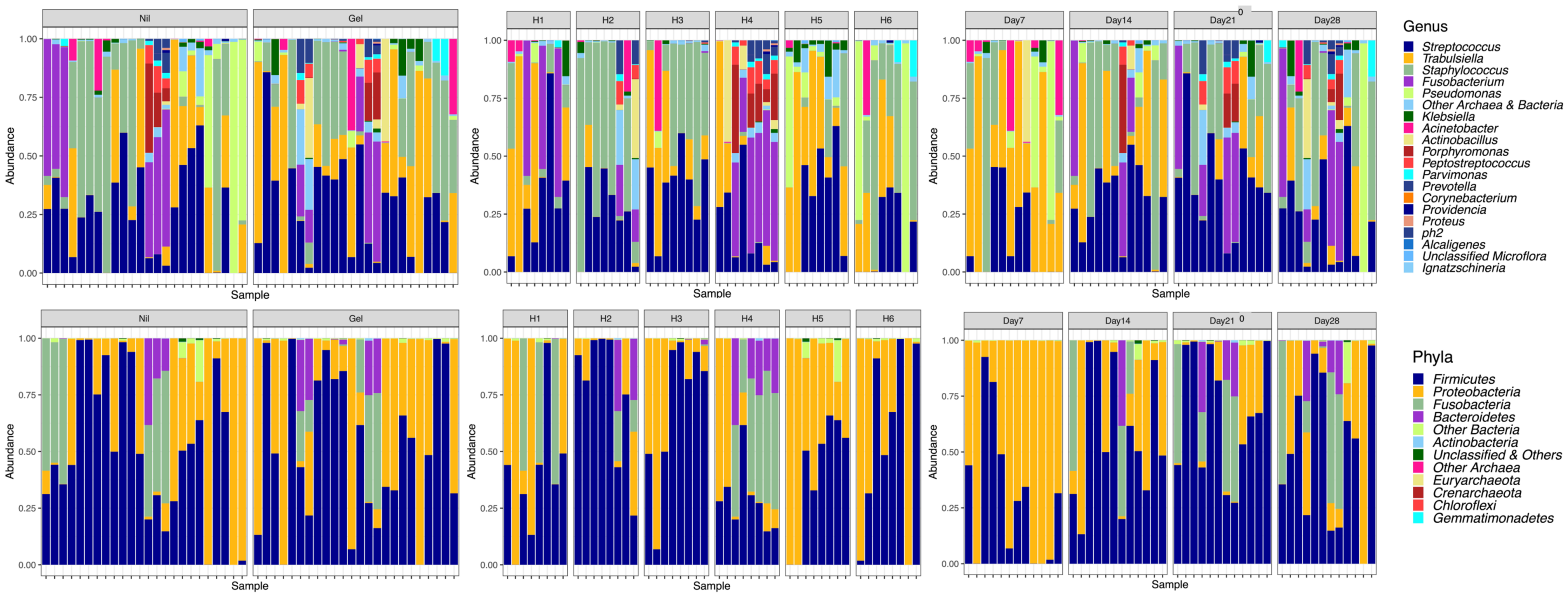
Relative abundances at the level of genus and phyla illustrated by Treatment (Nil; Gel), Horse (H1-H6) and Time (Day7-28).

The most commonly detected phyla were Firmicutes and Proteobacteria (Figure 3). At the genus level, *Staphylococcus* spp. (25.3%), *Streptococcus* spp. (24.3%) and *Trabulsiella* spp. (24.2%) followed by *Fusobacterium* spp. (8.7%) *Pseudomonas* spp. (3.5%), *Klebsiella* spp. (2.3%), *Actinobacillus* spp. (1.5%) and *Porphyromonas* spp. (1.18%) were most prevalent. Unclassified sequences amounted to 0.04 percent of the total number of reads. *Trabulsiella* spp. formed the dominant isolate early in wound healing while *Streptococcus* spp. and *Staphylococcus* spp. were most prevalent after 7 days (Table 1). With time, sample diversity appeared to increase (Figure 3). Differences in OTU proportions between treatment groups were significant for the genera *Actinobacillus* and *Pseudomonas* (Table 1). In the horse showing discordant wound healing (H4) sample microbiota were generally more diverse with *Fusobacterium, Porphyromonas and Peptostreptococcus* spp. being more prevalent compared to others (Figure 3).

**Table 1:** Proportions of OTU’s > 1% of all reads by “Time” and “Treatment.”

Operational Taxonomic Unit (OTU)						Percent of total number of reads					
						7-Days	14-Days	20-Days	28-Days	Treatment	
kingdom	phylum	class	order	family	genus					Gel	Nil
Bacteria	Proteobacteria	Gammaproteobacteria	Enterobacteriales	Enteriobacteriaceae	Trabulsiella	44.9	18.19	7.27	10.53	27.38	16.64
					Klebsiella	1.7	-	1.4	2.87	2	-
			Pseudomonadales	Pseudomonadaceae	Pseudomonas	10.3	1.69	-	-	1.08	7.65•
				Moraxellaceae	Acinetobacter	8.5	-	-	2.24	4.24	1.51
			Pasteurellales	Pasteurellaceae	Actinobacillus	4.03	-	-	4.76	3.96•	-
	Firmicutes	Bacilli	Bacillales	Staphylococcaceae	Staphylococcus	17	32.1	25.6	26.02	20.51	30.14
			Lactobacillales	Streptococcaceae	Streptococcus	12.7	30.29	40.37	22.28	28.07	23.26
		Clostridia	Clostridiales	Peptostreptococcaceae	Peptostreptococcus	-	-	2.49	1.58	1.15	-
				Tissierellaceae	Parvimonas	-	-	1.24	1.88	-	-
	Bacteroidetes	Bacteroidia	Bacteroidales	Porphyromonadaceae	Porphyromonas		3.28	3.36	4.51	2.2	2.72
				Prevotellaceae	Prevotella	-	-	1.9	-	1.2	-
	Fusobacteria	Fusobacteriia	Fusobacteriales	Fusobacteriaceae	Fusobacterium	-	10.36	12.78	17.45	5.16	13.54
Percent sum						99.13	95.91	96.41	94.12	96.95	95.46
Total number of reads						673,158	549,348	546,001	31,975	1,197,725	882,757

Differences in proportions that reached significance are marked by “•.”

#### 3.2.2 Alpha-diversity

**Figure 4:**
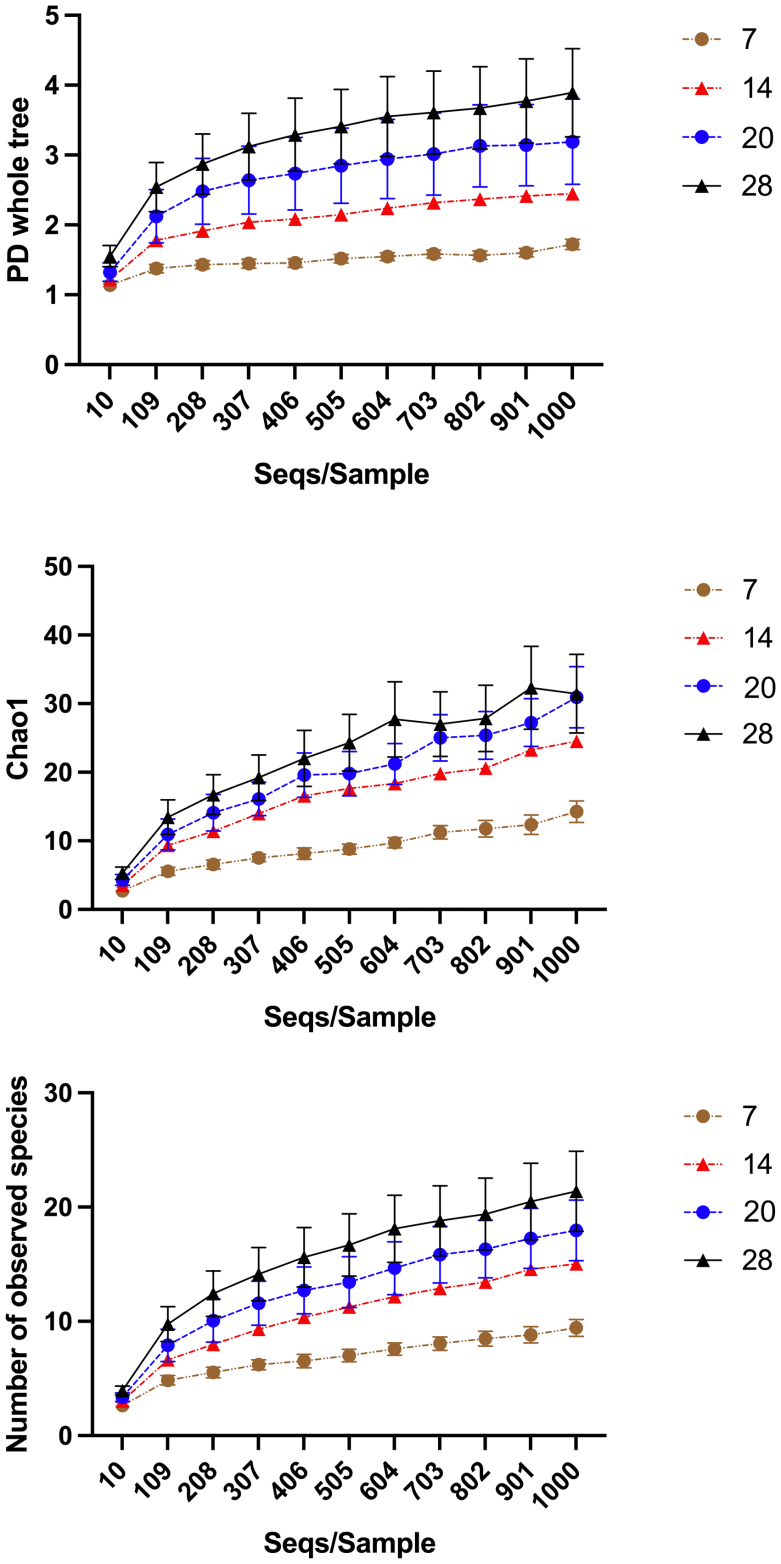
Alpha-diversity indices over time. “Horse” and “Time” had a significant effect on all alpha-diversity indices (*p* < 0.0001). As time progresses diversity indices of samples increases. The effect of “Horse” is illustrated in the Appendix.

“Treatment” had a significant effect on “Number of observed species” and “Chao1” (*p* < 0.001) but not “PD whole tree” (*p* = 0.108). “Chao1” and “Number of observed species” in primary wound dressings were lower when wounds were untreated (Table 2). “Horse” and “Time” had a significant effect on all alpha-diversity indices (*p* < 0.0001). There was marked variability between animals but H4 samples were most diverse (Appendix A) and diversity indices tended to increase for all animals as time progressed (Figure 4).

**Table 2:** Effect of treatment on alpha-diversity.

	PD whole tree		Chao1		N^o^ observed species	
	Gel	Nil	Gel	Nil	Gel	Nil
Median	1.794	1.748	12.983	9.500	9.600	7.100
IQR	1.145	0.637	13.828	12.733	8.250	7.000

Treatment had a significant effect on “N^o^ (number) of observed species” and “Chao1” (*p* < 0.001) but not “PD whole tree” (*p* = 0.108).

#### 3.2.3 Beta diversity

**Figure 5:**
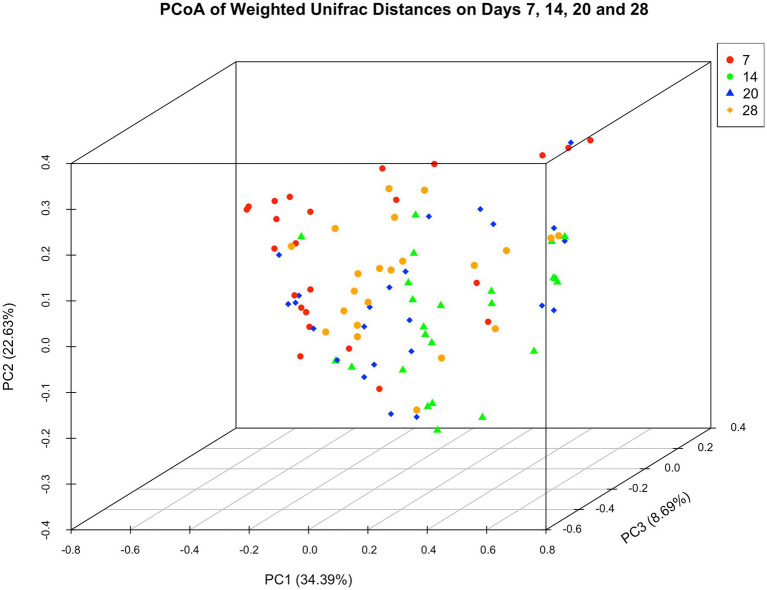
Principal coordinate analysis of weighted unifrac distances of microbial communities. Day 7 samples varied in their location from those of later time points.

Microbiota did not differ in dressings from wounds receiving different treatments (*p* = 0.384) but they varied between Day 7 and other time points (P_Day14_ = 0.0016; P_Day20_ < 0.001; P_Day28_ = 0.0009). Microbiota further differed between horses (P_H1vsH2_ = 0.0135; P_H1vsH4_ = 0.03; P_H1vsH6_ = 0.0163; P_H2vsH5_ = 0.0042; P_H4vsH2_ = 0.0002; P_H4vsH3_ = 0.0002; P_H4vsH5_ = 0.0006; P_H4vsH6_ < 0.0001; P_H6vsH5_ = 0.0251). Figure 5 illustrates the three main principal components as a function of “Time.”

### 3.3 Wound gel characterization

Aerobe and anaerobe culture of wound gel samples did not yield any bacterial growth, however 2, 6 and 7 OTUs were observed amounting to 876, 13,801 and 48,988 OTU reads from gel sample 1, 2 and 3, respectively. For sample 1, 2 and 3 the majority of reads (84 65 and 55% respectively) belonged to the genus *Streptococcus*. The second most prevalent genus was *Proprionibacterium* (Sample 1–15%) and *Kocuria* (Sample 2–20%; Sample 3–15%). Appendix B shows relative abundances in Gel samples.

### 3.4 Histology

#### 3.4.1 Descriptive findings

No microorganisms were observed on or under the wound surface or within the body of the granulation tissue in any of the biopsies studied. There was progressive re-epithelialization of wounds, changes in collagen staining patterns (from immature to mature), vascular development and organization, wound cellularity and matrix composition (from fibrin to collagen containing). These temporal changes were in line with progressive wound healing and consistent with a reduction in the histopathological characteristics of acute inflammation and the formation of disorganized and then organized granulation tissue.

#### 3.4.2 Quantitative analyses

With data pooled over time, treatment had no effect on the prevalence of PMNCs within tissues or the maturity of the vascular bed or collagen structures. With data pooled over treatments, time had a significant effect on the prevalence of PMNCs showing a progressive decline (*p* < 0.001; adjusted D7 vs. D42 comparison; *p* = 0.001). In analyses separated by treatment, a significant effect of time on the reduction of PMNCs was observed in both groups (P_Gel_ = 0.009; P_Nil_ = 0.012). However, in adjusted *post hoc* comparisons only untreated wounds showed a reduction in PMNCs in final biopsies (D7 vs. D42 comparison; P_Nil_ = 0.015; P_Gel_ = 0.254). No other significances were observed.

## 4 Discussion

In contrast to a previous report on the use of a 25% PG gel (21), an 80% formulation did affect equine wound healing in the current study. Treated wounds were consistently larger and relevant wound size reduction was observed later than in controls but effects were modest and unlikely to be of clinical relevance. Not unexpectedly wound size was correlated with total bacterial burden in primary wound dressings and codependent on “Time” and “Horse.” “Treatment” slowed the temporal decline in absolute abundances and increased alpha-diversity, but had no effect on beta diversity. Treatment delayed the natural decline in PMNCs during wound healing and representative of a prolonged inflammatory phase, facilitated the delayed healing rate. Our hypothesis that PG gel treatment changes microbial communities in primary wound dressings is thereby confirmed. However, there was no evidence that treatment affected ultimate wound healing outcomes.

In this investigation an 80% PG formulation was chosen as it provided the required solubility for a potential drug candidate. While PG in formulations with similar concentrations is classified as an inactive ingredient (19), subtle effects on the wound healing rate may relate to this concentration and direct effects on relevant cell function (20). However, as suggested by the presented data, the application of a topical wound formulation may result in greater wound associated bioburden in turn leading to the persistence of inflammatory cell infiltrates and a reduced healing rate. A similar observation was made when petrolatum, a different topical vehicle, was applied to experimental wounds, and culture dependent methods associated this intervention with greater bacterial growth and persistence of neutrophils in wound tissues (30).

As the equine cutaneous microbiome differs between seasons (31) and anatomical areas (12, 31, 32) it may not be surprising that prevalent genera in this investigation (*Staphylococcus*, *Streptococcus* and *Trabulsiella* spp) differed to those previously described (*Actinobacillus and Pseudomonas* spp) using the same experimental animal model (12). However, at the phylum level most prevalent OTUs were identical to the only other study on experimental equine wound healing (Proteobacteria, Firmicutes, Fusobacteria and Bacteroidetes) (12) and in a study of the equine cutaneous microbiome (Proteobacteria and Firmicutes) (31). A similar temporal pattern, i.e., a decline in the prevalence of Proteobacteria with concurrent increase in prevalence of Firmicutes and gradual appearance of members belonging to Fusobacteria and Bacteroidetes phyla was also observed (12). Taken together these similarities may suggest that microbiota recovery from primary wound dressings affords a relevant overview of wound tissue phyla without performing biopsies and the risk of disrupting normal wound healing processes. However, this data only provides preliminary insight until isolates from wounds and primary dressings are compared in the same wound environment thereby allowing a conclusive evaluation of this approach. As to the similarity of microbial communities in the context of time, only Day 7 samples showed some clustering which is likely explained by the relative lack of sample diversity and the relatively low abundance of *Streptococcus* spp. when compared to subsequent sampling time points.

Consistent with a previous report (12), observations varied widely between horses justifying the use of mixed model analyses to evaluate treatment effects with the animal declared a random effect. Interestingly when the individual animal effect was considered, it became apparent that alpha and beta-diversity indices for H4 samples were different to those of most other horses. Wound healing in H4 was characterized by marked hypergranulation, requiring the most extensive tissue debridement on Day 20. While we did not intend to explore the role of microbiota in delayed wound healing it should be noted that *Porphyromonas* spp. were only observed in H4 and *Fusobacterium* spp. were far more prevalent for this horse than in any of the other animals, except H1. However, Porphyromonas did not appear to play a pivotal role in the development of hypergranulation tissue as the wound with the greatest formation was not associated with this genus (Appendix C). Members of the phyla Bacteroidetes and Fusobacteria play an important role in oral health and periodontal disease across species (33, 34). Consequently they are also prevalent pathogens in human and animal bite injuries (35). As sampling sites remained bandaged and protected from self-contact and potential oral transmission, environmental/fecal origin of aforementioned phyla is likely more plausible (36, 37).

While there is some evidence to associate anaerobic infections of other organ systems with poorer outcomes (38) and *Porphyromonas and Fusobacterium* spp. with complicated bovine foot lesions (39, 40), the authors are not aware of comparable evidence in chronic equine wounds although others have speculated on the relevance of *Fusobacterium* spp. in equine distal limb wounds (12). In people on the other hand, their putative role is much clearer as anaerobe microbial communities have been associated with longer wound duration and ulcer depth (41, 42).

In this and other investigations (34) greater alpha-diversity indices were associated with negative biological effects. In contrast, others have associated lower values with disease and disease severity both in horses (15, 43) and people (42, 44) suggesting that the biological relevance of diversity indices are yet to be fully understood (6). Recent evidence suggests that rather than diversity, dynamic stability of microbial communities may be indicative of a host’s immune response and play a more relevant biological role (2). An effect of microbial stability could not be observed in the available data set (Figure 3), however the last sampling was performed prior to the completion of wound healing which may have prevented the opportunity for making this observation.

Propylene glycol gel treatment delayed the temporal decline in PMNCs in tissues and absolute microbiota abundances in dressings, increased sample richness in dressings, and slightly delayed wound healing without causing an obvious effect on final wound outcomes. Our results suggest that this effect was not driven by live microbial contamination of gels given the negative culture results and the histologic absence of bacteria. Nevertheless, a small proportion of OTUs may have been attributable to gel contamination given results with culture independent detection methods. Instead of bacterial contamination, we argue that promotion of mild inflammation and a more occlusive wound environment following gel application facilitated survival and retention of microbial communities and/or the formation of biofilms. Biofilm formation plays an important role in delayed wound healing in human (45) and equine wounds (32, 46), but this aspect was not explored in the current investigation to validate assumptions. Other shortfalls in this investigation relate to the absence of pre-wounding samples for next generation sequencing which would have afforded greater comparative insight. Not necessarily in the context of short falls, but the impact of sampling sites on data validity required further consideration in post experimental analyses. While the observed complicated wound healing of proximally positioned wounds was attributed to bandage failures, others have had similar experiences and established that topographical site differences exist (47). Consequently, it was decided to exclude proximal limb wound data all together. Lastly, it is to be emphasized that as biopsies were only obtained from distal limb wounds, histologic observations do not directly correlate with middle wound data. Given that repeated biopsy procedures would have invariably altered normal wound healing processes and the wound microbiome, this compromise was considered essential.

## 5 Conclusion

Topical wound applications have the potential to alter resident microbiota. Stringent controls are required to differentiate the effects of formulations from those of their therapeutic constituents. While the clinical treatment potential for microbiota manipulation is yet to be determined, survey of primary wound dressings allows generation of meaningful data warranting further research to establish if it also permits temporal follow-up without invasive sampling.

## Data availability statement

The original contributions presented in the study are publicly available. This data can be found here: https://www.ncbi.nlm.nih.gov/bioproject/PRJNA1019907.

## Ethics statement

The animal study was approved by Animal Ethics Committee (A18081) of Charles Sturt University, NSW, Australia. The study was conducted in accordance with the local legislation and institutional requirements.

## Author contributions

RL: Conceptualization, Data curation, Formal analysis, Investigation, Methodology, Project administration, Resources, Supervision, Writing – original draft, Writing – review & editing. SR: Conceptualization, Data curation, Formal analysis, Investigation, Methodology, Project administration, Writing – review & editing, Supervision. CB-N: Data curation, Formal analysis, Investigation, Writing – review & editing. SDP: Data curation, Formal analysis, Methodology, Writing – review & editing. SP: Formal analysis, Methodology, Writing – review & editing. TDR: Conceptualization, Formal analysis, Funding acquisition, Investigation, Methodology, Project administration, Resources, Writing – review & editing.

## Footnotes

^1^ https://github.com/lh3/seqtk

## Supplementary material

Supplementary Appendix A Species richness in samples stratified by “Horse”.

Supplementary Appendix B Relative abundances in Gel samples.

Supplementary Appendix C Day 14 wound images and corresponding relative microbial abundances in primary wound dressings.
