# Supplementary figures and images for: Wound healing of experimental equine skin wounds and concurrent microbiota in wound dressings following topical propylene glycol gel treatment
Source: Front Vet Sci. 2023 Dec 14;10:1294021. doi: 10.3389/fvets.2023.1294021 (PMC10752953; doi:10.3389/fvets.2023.1294021)

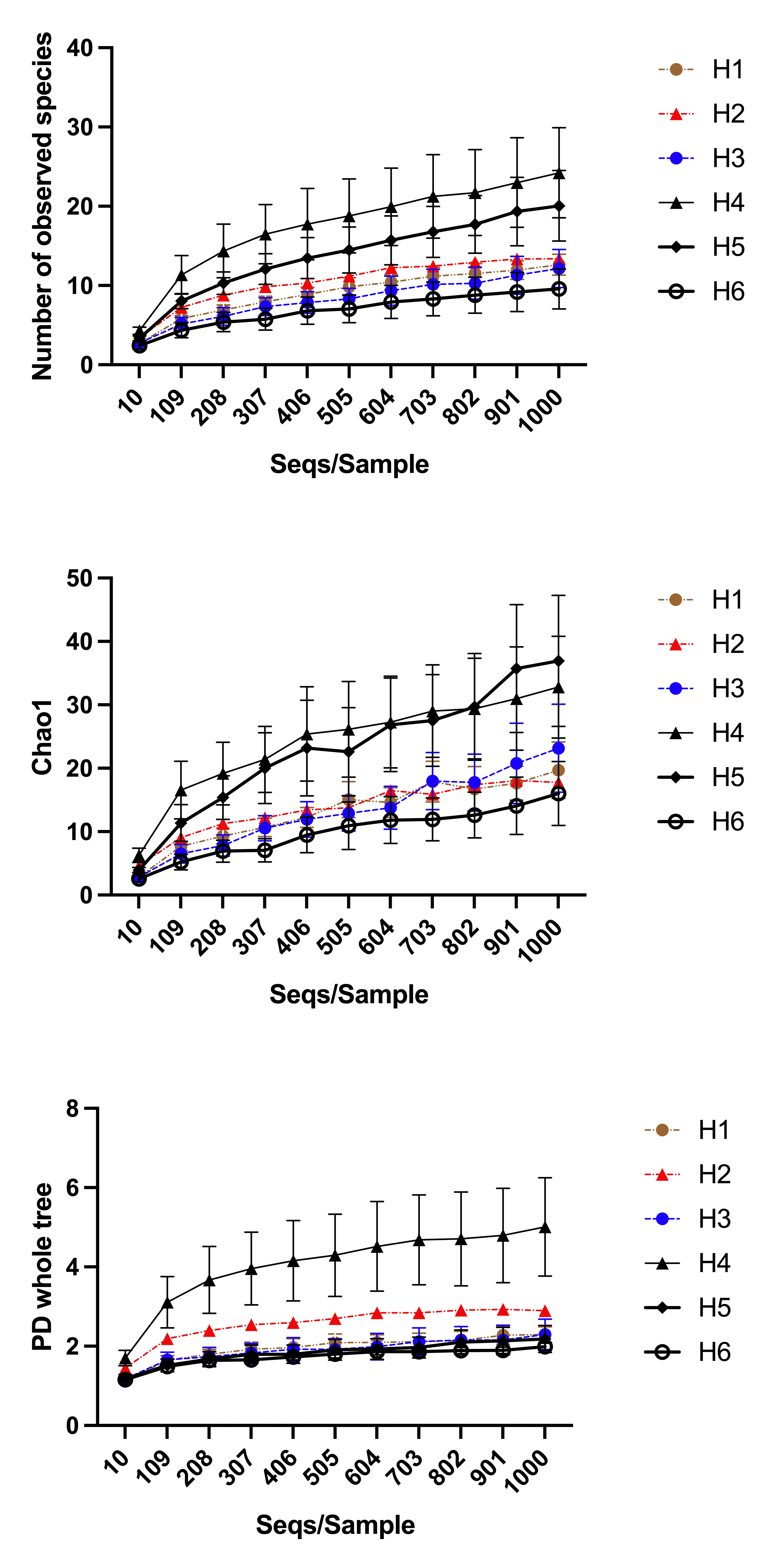

Supplement: Supplementary Appendix A — Species richness in samples stratified by “Horse”. [file Image_1.TIFF]

# Appendix B

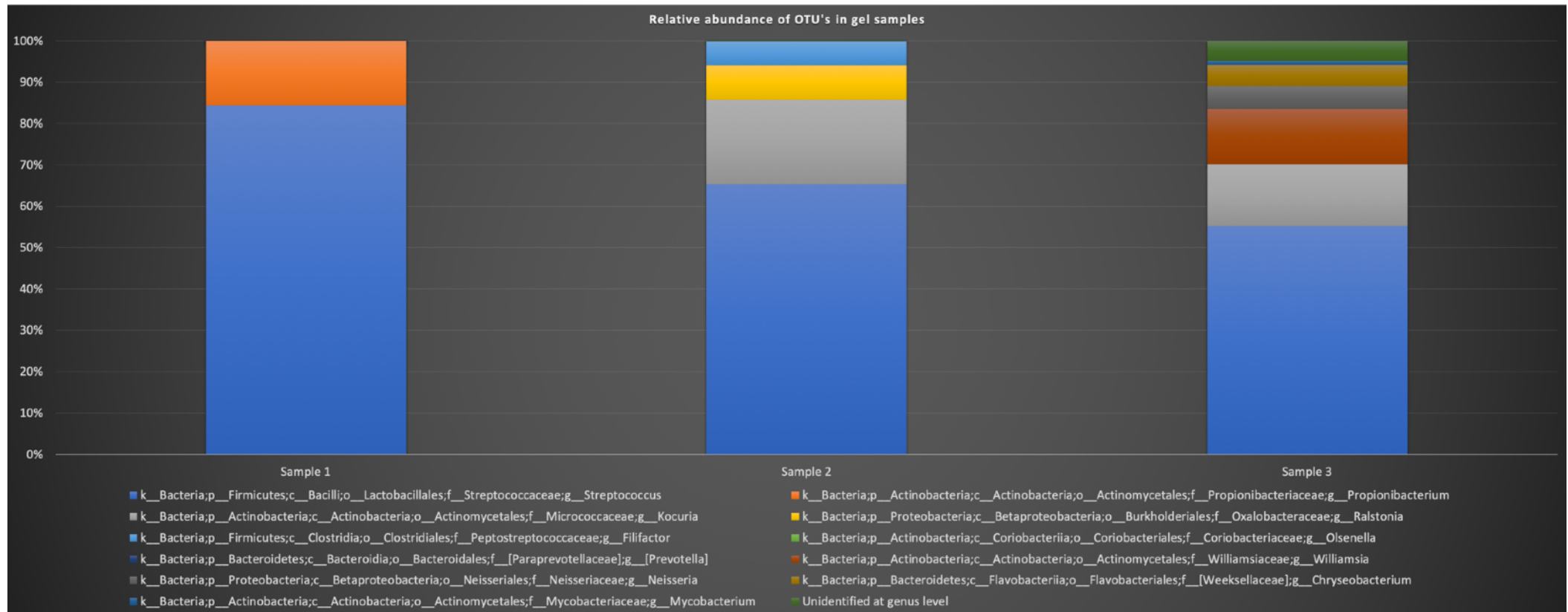

Supplement: Supplementary Appendix B — Relative abundances in Gel samples. [file Image_2.PDF]
